# Supportive Care of Hematopoietic Stem Cell Donors

**DOI:** 10.46989/001c.92460

**Published:** 2024-02-20

**Authors:** Wolfgang P Rennert, Jenna Smith M, Katie A Cormier, Anne E Austin

**Affiliations:** 1 Blood and Marrow Collection Program MedStar Georgetown University Hospital; 2 Stem Cell Transplant and Cellular Therapy Vanderbilt University Medical Center

**Keywords:** supportive care, hematopoietic stem cell donation, peripheral blood stem cell, bone marrow harvest

## Abstract

Supportive care needs for hematopoietic stem cell recipients have been studied. Less is known about the care needs of stem cell donors. Care challenges arise at donor selection, preparation for the donation, the donation procedure and the immediate and long-term after-care. Care needs were analyzed for 1,831 consecutive bone marrow and peripheral stem cell donors at MedStar Georgetown University Hospital between January 2018 and August 2023 in support of a review of the current literature. During the selection, related donors may experience psychological pressures affecting their motivation, while donation centers may be willing to accept co-morbidities in these donors which might preclude donation in unrelated peers. For bone marrow donations, it is important to select donors not only according to optimal genetic matching criteria but also according to suitable donor/recipient weight ratios, to facilitate sufficient stem cell yields. During the donation preparation phase, side effects and complications related to stem cell stimulation must be anticipated and managed for peripheral cell donors, while the pros and cons of autologous blood donation should be evaluated carefully for bone marrow donors. The stem cell donation procedure itself carries potential side effects and complications as well. Peripheral cell donors may require a central line and may encounter hypocalcemia, thrombocytopenia, and anemia. Bone marrow donors face risks associated with anesthesia, blood loss and pain. Post-procedure care focusses on pain management, blood cell recovery and the psychological support necessary to regain a high quality-of-life existence. Hematopoietic stem donors are giving part of themselves to save another’s life. They deserve comprehensive supportive care to accompany them throughout the donation process.

## Introduction

Allogeneic hematopoietic stem cell transplantation is a potentially curative treatment for a variety of malignant and non-malignant disorders. The supportive care of hematopoietic stem cell transplant recipients has been studied extensively. It includes the physical and psychological preparation of the prospective stem cell recipient for the transplant, the management of co-morbidities, the alleviation of transplant-related symptoms and toxicities, the prevention and management of acute and long-term complications, and the preparation for the reintegration into a high quality of life existence afterwards.^1–3^ Diligent monitoring, cooperation with the recipient’s family members, and collaboration with the multidisciplinary transplant team can improve post-transplantation outcomes.^2,4,5^

Less is known about the best practice of supportive care for stem cell donors. Psychological care needs may arise as early as at the point of deciding to become a donor – particularly for related donors.^6^ In the pre-donation period, peripheral blood stem cell (PBSC) donors require granulocyte colony-stimulating factor (G-CSF) mobilization, which may be associated with complications. Bone marrow donors may be asked to donate a unit or two of blood to be re-infused after the procedure. Central venous catheters may be required for some peripheral stem cell donations. Bone marrow donors undergo a surgical procedure under general or regional anesthesia with potential complications. These include pain, hypovolemia, thrombocytopenia, and hypocalcemia.^7^ Long-term care needs include the psychological support of related donors whose transplants failed to secure a loved-one’s survival.^8^

This review describes and discusses supportive care needs for stem cell donors across the donation process from selection, through the pre-donation preparation period and the stem cell donation procedure, to the immediate and long-term recovery phase. The review is supported by data from the MedStar Georgetown University Hospital Blood and Marrow Collection Program (MGUH-BMCP) (Table 1).

**Table 1.** baseline data from reviewed donors at MGUH-BMCP for marrow donors (HPC-M) between January 2018 and August 2023 and PBSC donors (HPC-A) between January 2020 and August 2023.

	HPC-M (2018 – 2023) n = 1132 mean (range)	HPC-A (2020 – 2023) n = 699 mean (range)
Age (years)	29.2 (18-58)	30.6 (18 – 71)
Male/female (n)	654 / 478	362 / 335
BMI (kg/m^2^)	27.4 (17.3 – 42)	28.2 (17.5 – 49)
Donor/recipient weight ratio	2.6 (0.4 – 20.4)	1.4 (0.4 – 22.2)
estimated blood volume (mL)	5,006 (2,649 – 8,172)	
Hb before (g/dL)	14.2 (9.9 – 19.2)	14.1 (9.9 – 18.1)
Hb after (g/dL)	10.9 (5.8 – 16.1)	12.6 (8 – 16.3)
Platelets after procedure (x10^9^/L)		126 (52 – 313)
Volume harvested (mL)	1,187 (400 – 1,873)	
PBSC volume processed (L)		17.3 (10 – 30)
% blood volume harvested	24.2 (6.6 – 43.1)	
TNC/kg recipient weight collected (x10^8^)	6.88 (1.56 – 39.7)	
CD34/kg recipient weight collected (x10^6^)		10 (1.1 – 89.7)
Total TNC collected (x10^8^)	261 (72 – 654)	
Total CD34 collected (x10^6^)		698 (99 – 2,893)
Auto transfusion received	225 / 1131	

## Donor selection

Historically, successful stem cell transplant has relied on a perfect match of human leucocyte antigen (HLA) patterns between donor and recipient. Modern chemotherapeutic regimens allow the match of sub-optimal HLA types, including haplo-identical HLA patterns found in family members of patients in need of stem cell transplants.^9^ This has significantly increased the potential pool of accessible stem cell donors. Nevertheless, donor factors beyond a close HLA-match can affect and challenge a successful transplant outcome.

**Figure 1.**
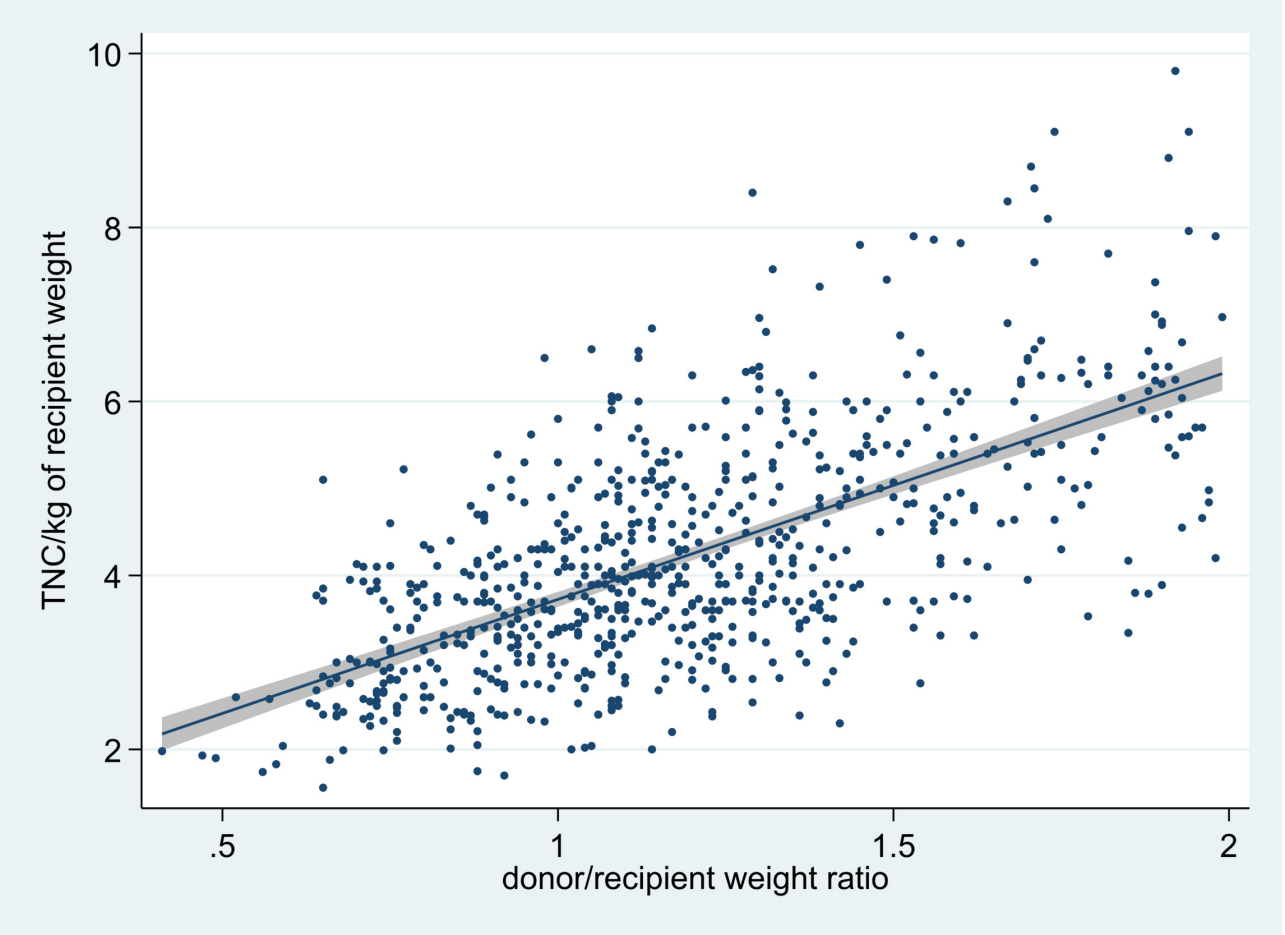
TNC/kg of recipient weight for donor/recipient weight ratios up to 2 including line of best fit with 95% confidence interval.

At the MGUH-BMCP, we established that total nucleated cell (TNC) yields of more than 2x10^8^/kg of recipient weight could only be provided with 95% confidence when the bone marrow donor’s weight was at least 80% of the recipient’s^10^ (Figure 1). In some circumstances, donor co-morbidities that preclude donation in unrelated donors might be considered acceptable in the case of related donors, particularly when the cell donation is performed at the same site of the respective transplant,.^11–13^

Related donor motivation may be affected by external factors including pressure from family members, religious beliefs or perceived social obligation,^14^ as opposed to the more internal motivators reported by unrelated donors like altruism, a sense of duty, and the ability to form a strong emotional connection with an unknown recipient.^15,16^

Recommendations for supportive care during donor selection should therefore include a separation of stem cell collection teams from stem cell transplant teams,^11^ strict adherence to transplant suitability guidelines for related and unrelated donors as directed by the National Marrow Donor Program (NMDP) and the Centers for International Blood and Marrow Transplant Research (CIBMTR),^12,13,17,18^ a clear evaluation of donor motivation to eliminate the influence of external pressure on the decision making process,^6,8^ and the selection of donors who match genetically and in size with their respective stem cell recipients.^10,19^

## Pre-procedure preparation

The pre-procedure preparation carries challenges for both PBSC and bone marrow donors. The majority of PBSC donors experience mild to moderate bone pain, headaches and discomfort during their five-day G-CSF regimen,^20,21^ while bone marrow donors may be asked to donate one or two units of blood in preparation for the donation. Serious side effects are rare.

Among 911 PBSC donors at MGUH-BMCP, two developed an anaphylactic reaction to the G-CSF product *Filgrastim*, necessitating the interruption of PBSC preparation and the conversion to a bone marrow donation. In five cases, *Filgrastim* protocols were shortened due to prohibitive side effects (2 because of headaches, 1 because of diarrhea, 1 because of vomiting, and 1 because of an allergic skin reaction). One donor experienced a splenic rupture on the day after the last *Filgrastim* application and the PBSC donation, requiring the surgical placement of a coil to stop further bleeding. In another case a back-up donor had to be activated because the PBSC donor experienced serious diarrhea and vomiting on day 4 of *Filgrastim*.

The use of preoperative autologous blood donation for bone marrow donors has changed over the years. Associated risks include bacterial contamination, erroneous use of incompatible blood units, hemolysis, and wastage of unused blood products.^22,23^ Incomplete blood cell recovery between collecting the autologous unit and bone marrow harvest may also neutralize potential benefits.^24,25^

**Figure 2.**
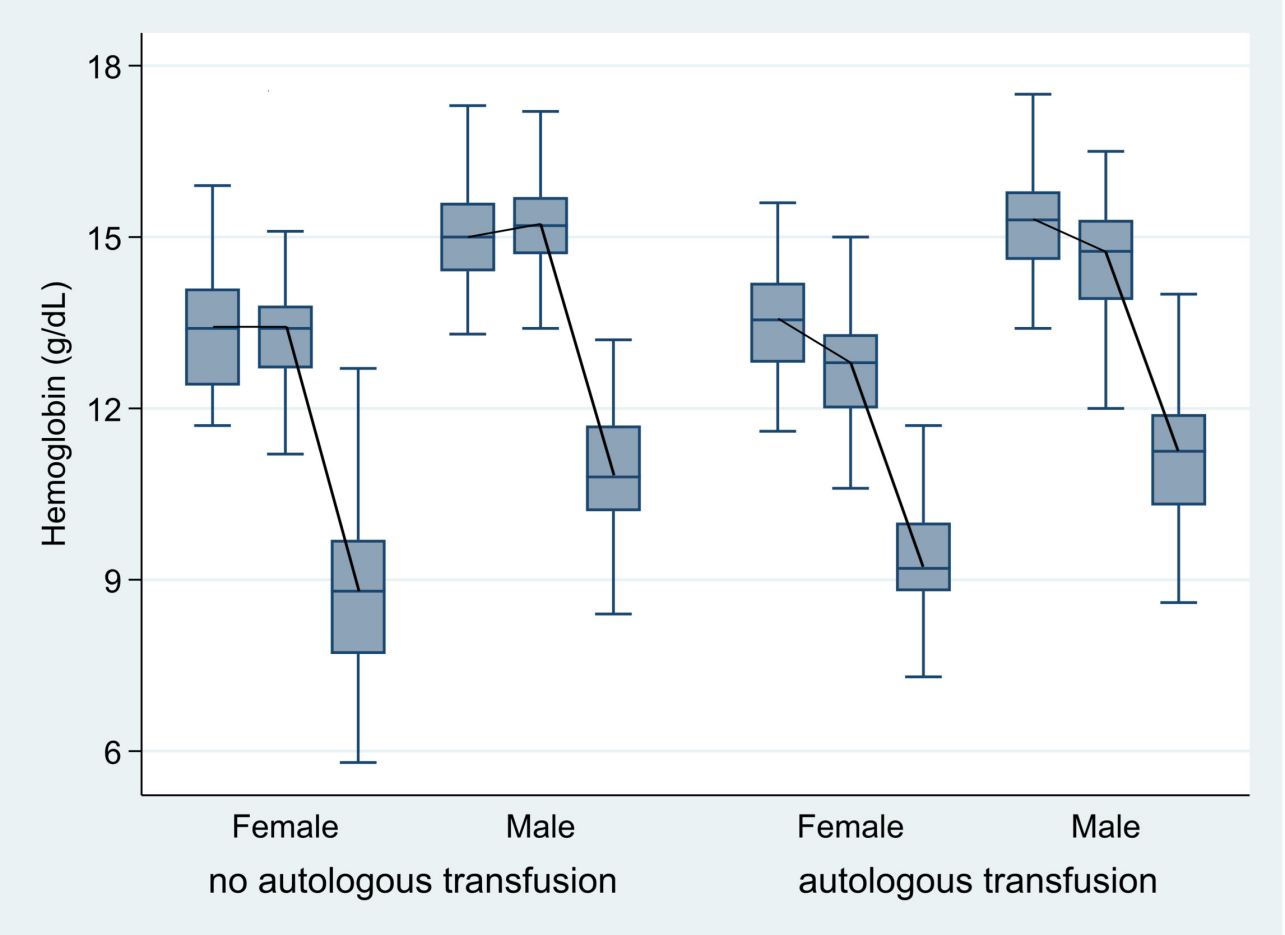
Hemoglobin levels at the time of preoperative autologous blood donation (box 1), at the time of bone marrow donation (box 2) and after bone marrow donation (box 3) for female and male donors with and without an autologous unit of blood.

At the MGUH-BMCP, we noticed that hemoglobin levels did not recover fully after autologous blood donation by the time of the bone marrow harvest (Figure 2). We observed critical hemoglobin drops to levels requiring an allogeneic transfusion in three female donors without autologous blood donation (Figure 2). These donors had donated the maximum of 20mL/kg accounting for more than 33% of estimated total blood volume. In response, the MGUH-BMCP team has since decided to limit the maximal marrow donation volume in female donors to the lesser of 18mL/kg, 30% of estimated blood volume or 1,500 mL, while maintaining maximal donation levels for male donors at the lesser of 20mL/kg, 33% of estimated blood volume or 1,500mL total volume. The use of preoperative autologous blood donation has since been discontinued at the MGUH-BMCP, in line with recommendations from elsewhere.^26–29^

Recommendations for supportive care during the pre-procedure preparation phase should include careful preparation of PBSC donors to anticipate complications related to G-CSF, and the management of pain, nausea, discomfort, and allergic manifestations of *Filgrastim* applications. For bone marrow donors, a careful determination of maximal donation volume should be established, and iron deficiency anemia should be corrected prior to the donation.^30,31^

## Stem cell collection with associated short-term complications

For PBSC donors, the actual stem cell collection carries different complications from the preparation with G-CSF. Problems may arise when venous access challenges require a central venous line (CVL). CVL placement needs to be performed by experienced staff to minimize the risk of bleeding, pneumothorax, pulmonary or cardiac injury, blood stream infections, and thromboembolic events.^32–34^

Hypocalcemia, thrombocytopenia and to a lesser extent anemia are also important complications of PBSC that require appropriate supportive care. Citrate infusion during standard volume apheresis procedures leads to a decrease in ionized serum calcium concentrations between 35 and 56%.^35^ At MGUH-BMCP, intravenous calcium gluconate is supplemented at 1.5 g per hour of apheresis. Smaller donors receive a citrate-heparin combination supplemented by calcium gluconate at 0.75 g per hour.

Complications related to G-CSF application and apheresis itself, such as bone pain and fatigue, are usually mild and transient.^36,37^ Older donors, females and donors with preexisting medical conditions may experience symptoms for longer periods, but complications lasting more than a month are unusual for PBSC donors.^20,38^ Serious side effects for PBSC donors are rare. Among the 911 PBSC donors at MGUH-BMCP during the observation period of 2018 through 2023, 36 required CVL placement. Two of these experienced significant bleeding after CVL removal. One donor developed thrombocytopenia without bleeding, and one donor experienced hypocalcemia with trismus and carpal spasms.

Complications of bone marrow donation relate to the procedure itself (bone pain in hips and lower back) and to the anesthesia (headache and throat pain). At MGUH-BMCP, more serious anesthesia-related complications included bronchospasm in one donor during intubation. In this case, the bone marrow collection was aborted, and the collection was converted to PBSC after a shortened G-CSF stimulation period. One donor experienced a broken tooth during intubation, and one donor suffered from atrial fibrillation during the procedure. This donor required cardioversion under anesthesia on the day following the donation.

Complications relating to the bone marrow harvest are typically mild and last for up to three weeks. Symptoms beyond one month duration are rare.^20,36–38^ At MGUH-BMCP, 1132 bone marrow collections were performed between 2018 and August 2023. Seven donors experienced pain beyond one month duration. All had resolved by 6 months. One donor reported having suffered a labrum tear in the hip that required surgical repair. Four donors became hypotensive during the procedure and required fluid resuscitation with colloid fluid infusions and inotropic support. Three donors required an allogeneic transfusion after the procedure. One donor experienced a vasovagal syncope on the day following the procedure.

**Figure 3.**
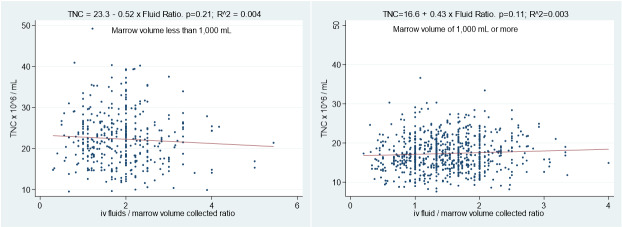
correlation between TNC density per mL of harvested bone marrow and intraoperative fluid volumes given during the procedure in relation to marrow volume collected for small (left) (p=0.21) and large (right) (p=0.11) bone marrow stem cell harvests.

The supportive care for PBSC donors during collection should focus on the prevention and management of hypocalcemia, hypovolemia, and thrombocytopenia. Great care should be dedicated to the placement of adequate peripheral venous access by expert phlebotomists to minimize the need for CVL placement. Supportive care for bone marrow donors should focus on safe anesthesia practice and intravenous fluid replacement to prevent intraoperative hypotension and maintain adequate circulation.^39,40^ Proceduralists should not restrict fluid replacement to increase total nucleated cell (TNC) density in the harvest product. At MGUH-BMCP, no significant correlation was noted between TNC density and fluid replacement volume (Figure 3). The harvest marrow volume withdrawn is replaced with crystalloid intravenous fluid in a 2:1 ratio during the procedure followed by another aliquot of post-operative fluid.

The supportive care of bone marrow donors in the immediate post-operative period focuses on pain management, wound care and the correction of anemia. Donors should avoid lifting heavy objects beyond 20 pounds for 2 weeks. Non-steroidal pain relievers should be avoided for 3 days following the procedure to prevent subcutaneous bleeding at the harvest sites. At MGUH-BMCP, bone marrow donors receive 150 mg of a polysaccharide iron complex daily for a month following the procedure to enhance hemoglobin recovery.^41^ Care should be taken to ensure that hemostasis has been achieved at the harvest incision sites and the donor has been educated on dressing care prior to discharge.

No clear guidelines exist for the use of perioperative antibiotic prophylaxis for bone marrow donors. Single-dose intravenous antibiotic application at the beginning of surgical joint or spine procedures has been established as efficacious to reduce surgical site infections.^42,43^ At MGUH-BMCP, 198 bone marrow harvests were performed between January 1^st^ and August 31^st^, 2023. Of these, 158 were performed with a single dose of intravenous clindamycin at the beginning of the procedure, and 40 without antibiotic prophylaxis. Clindamycin was chosen because of good coverage of gram-positive organisms typically residing on the skin, good bone penetration, and low risk of allergic or anaphylactic complications in the marrow recipient.

None of the bone marrow donors suffered from tissue contamination or infection. Equally, none of the donors suffered from potential clindamycin side effects such as the development of pseudomembranous colitis. Nine of 158 marrow products (6%) collected from donors who had received clindamycin showed contamination with skin related bacteria, while 9 of 40 marrow products (22.5%) of donors without antibiotic prophylaxis were contaminated. The chi-square product of this difference was 10.9057 with a significance of p<0.001. Consequently, at MGUH-BMCP, the use of single-dose intravenous clindamycin at the beginning of the procedure has been established as the standard of care.

## Long-term complications

The median recovery time for PBSC donors is typically about one week, while bone marrow donors report pain and discomfort for about three weeks following the donation.^20^ Longer recovery times are unusual but tend to occur in specific donor populations. Donors older than 40 years of age and donors with co-morbidities are more likely to report persistent pain at 1, 3 or 12 months after the procedure, or a failure to return to a pre-donation level of wellness.^44^ Equally, longer recovery times are experienced by donors who report psychosocial challenges in their health-related quality of life surveys beforehand.^45^ Particularly, related donors may describe a failure to return to a baseline quality of life or protracted discomfort twelve months after the donation.^8,46,47^

The supportive care for donors at this stage should use a multi-disciplinary approach with a focus on quality-of-life recuperation. Physical therapists, social workers and psychotherapists are tasked to collaborate with primary care teams to guide donors back towards a high quality-of-life existence.

## Conclusion

Supportive care needs for stem cell donors arise at all stages of the donation process. While physical care needs related to G-CSF application and the immediate side effects of stem cell donation itself are typically addressed by stem cell collection centers, more emphasis needs to be placed on the mental health care needs of donors aiming at a rapid return to a high quality of life existence after the donation process. Standards of procedure for supportive care strategies like the use of autologous transfusion or the prevention of product contamination with skin bacteria during the donation procedure require review and update by collection centers, based on data driven analysis of best practices. Individuals who give part of themselves to save another’s life deserve optimized and comprehensive care throughout the donation process.
